# Introduction of a mathematical model for optimizing the drug release in the patient’s body

**DOI:** 10.1186/2008-2231-22-1

**Published:** 2014-01-03

**Authors:** Mohammad Reza Nabatchian, Hamid Shahriari, Mona Shahriari

**Affiliations:** 1 Department of Industrial Engineering, K.N. Toosi University of Technology, No 7, Pardis St., Mollasadra Ave., Tehran, Iran; 2 Department of Dermatology, University of Connecticut Health Center, Farmington, CT, USA

**Keywords:** Drug release, Time-oriented quality characteristic, Parameter design, Desirability function, Release profile

## Abstract

**Background:**

Drug release in a patient’s body is of particular interest to the pharmaceutical industry. One of the most essential types of drug release is the gradual release based on a behavior, which is called a profile or modified release. The investigation of the time-oriented quality characteristic is one of the newest topics in the area of product design. There are already several approaches addressing this issue. In this paper, a mathematical model is proposed to find the suitable values of the controllable factors in a drug to achieve the profile of the drug release in the patient’s body.

**Results:**

The proposed method has several advantages over the existing methods.

**Conclusion:**

The authors feel that by adjusting the control factors during the production process the drug release profile become closer to the reference profile.

## Introduction

The amount of time it takes a drug to release in a patient’s body as well as the time it takes to exert its effects on the target organ are very important factors used to measure the effectiveness of a drug. If this releasing manner is not based on a pre-defined profile, it may cause a reduction of curative properties of the drug and can even have some negative effects on the patient’s body. Similarly, in the area of quality engineering, the time-oriented quality characteristics are also assessed. The time-oriented profile of the quality characteristic is specified and the aim of the designer is to find the predefined profile with minimum deviation from the target. The quality characteristics are then monitored using the defined profile. In this study, we aim to establish a logical relationship between these two areas and to apply a mathematical modeling approach to investigate the drug release problem in pharmaceutics. In this paper some basic definitions of drug release and quality engineering are presented and then we introduce the four existing approaches for these types of problems and their deficiencies. The proposed method is presented in the next section. Several examples are provided to evaluate the suggested model and in the final section, the conclusions are made.

### Definitions

In this section some of the basic terms included in the paper are defined to familiarize the reader with the concepts of the discussion.

### Drug release

**Figure 1:**
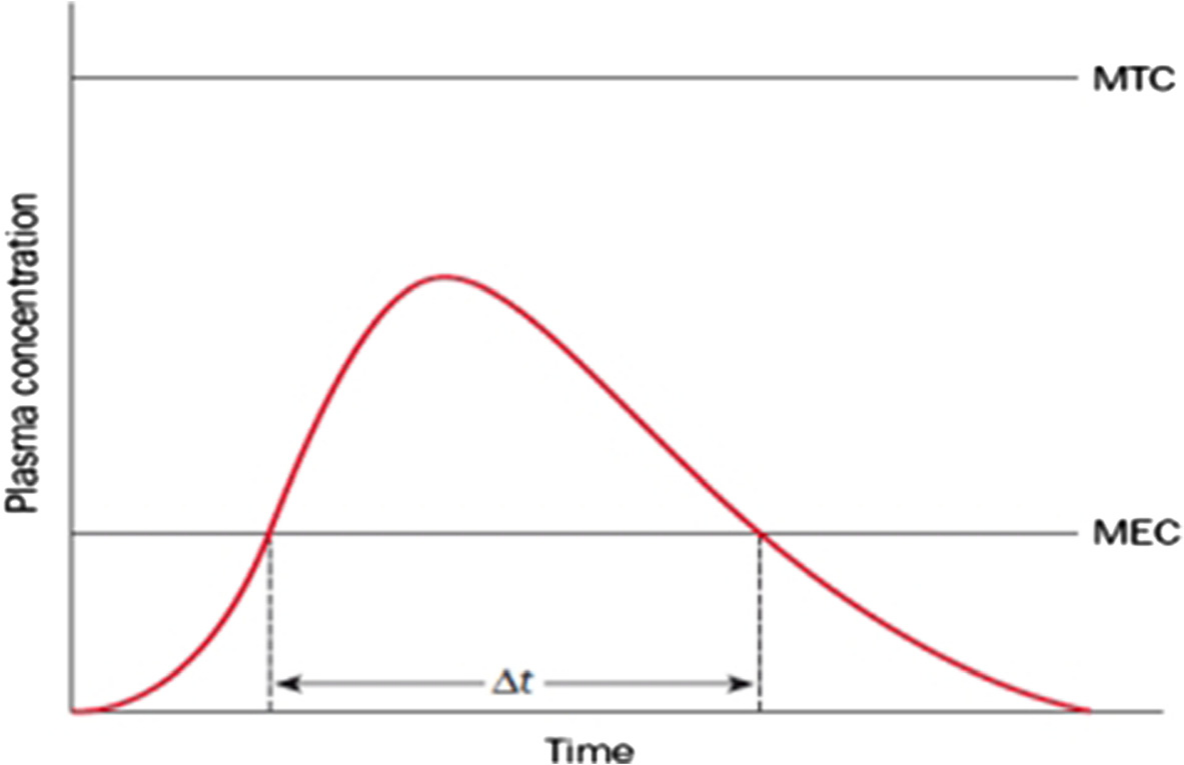
**Plasma concentration versus time profile**[1]**.**

Drug release is an important stage in the drug life cycle. When the drug is released based on a pre-defined profile, it is more effective on the patient’s body. One of the most applicable approaches for measuring the amount of released drugs is to measure the plasma concentration of the drug. The drug is considered effective when the plasma concentration is somewhere between minimum effective concentration (MEC) and minimal toxic concentration (MTC) as is shown in Figure 1[1-3].

Drugs are usually classified based on the drug release mechanism as follows:

Immediate release drugs: In this group, the drug is quickly released in the body. This is particularly suitable for drugs that need to take affect rapidly such as painkillers [1,4].

**Figure 2:**
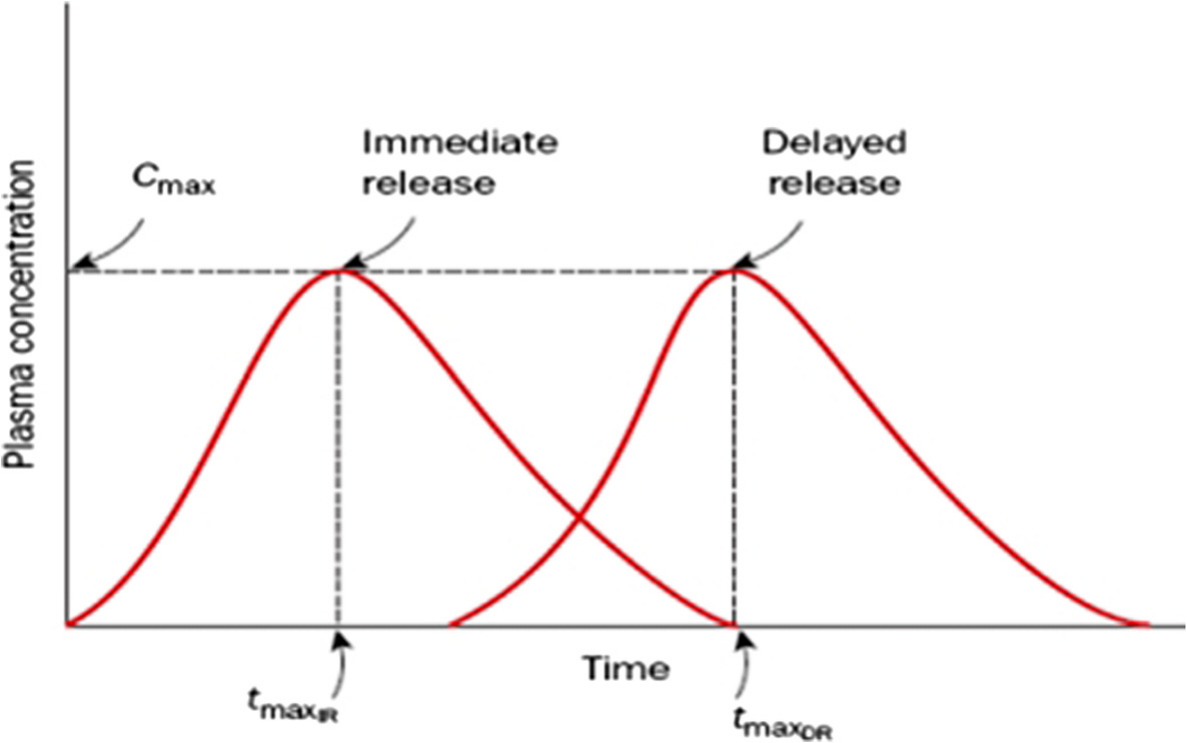
**Plasma concentration versus time profile for an immediate release drug and a delayed release drug**[1]**.**

Modified drug release: In this case by using the pharmaceutical techniques, the time, the amount and the target organ for the drug release is determined. The delayed release and extended release are the methods being used. In the delayed release the drug is released after a pre-determined delay. Figure 2 shows the plasma concentration for this modified release method [1,4].

**Figure 3:**
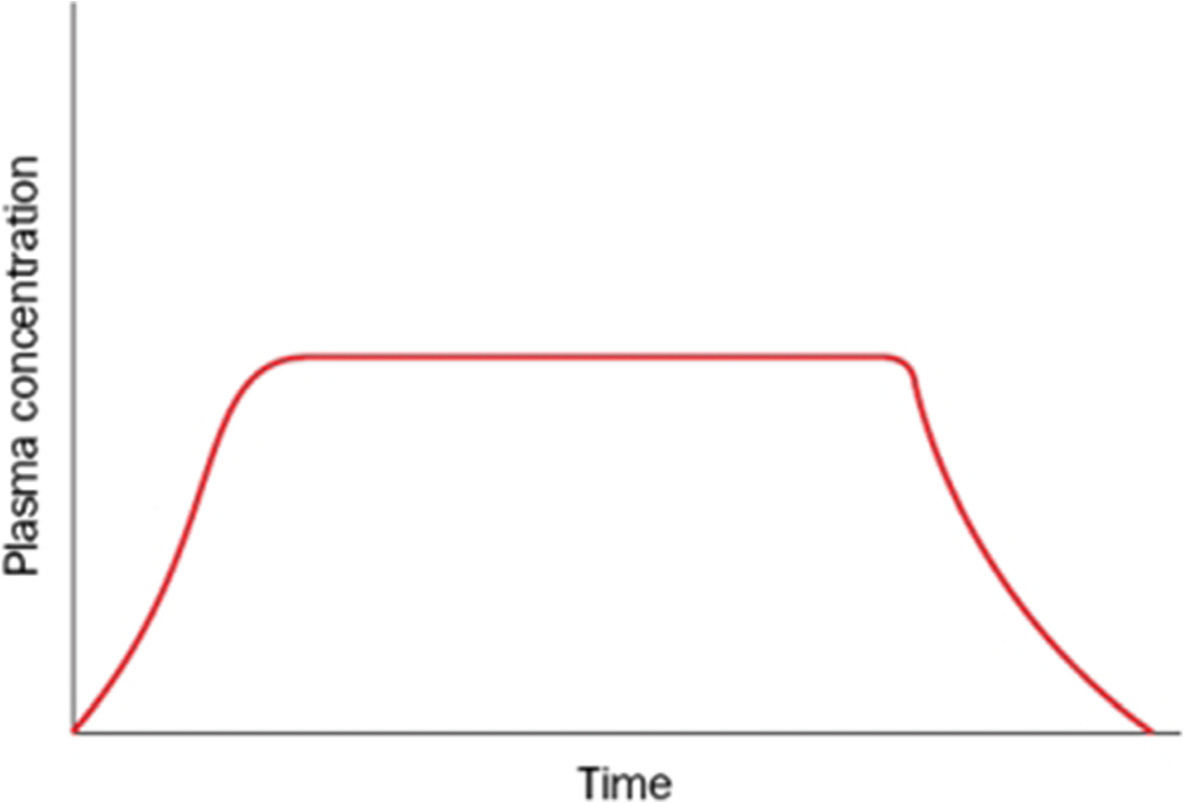
**Plasma concentration versus time profile for a controlled release formulation**[1]**.**

In the extended release technique, the drug is released gradually over a longer period of time. It is classified into two categories: sustained release and controlled release. In sustained release, the drug is released continuously with a constant rate. In controlled release, the drug is released intelligently so that the concentration remains almost constant in the body. Figure 3 shows the plasma concentration when using this method of drug release [1,5].

### Time-oriented quality characteristics

There are several definitions of the quality characteristics in the quality management literature. The most comprehensive of them is the degree of adaptability of the quality characteristic by the user’s requirements [6]. Furthermore, the design phase is the principal stage of a product life cycle, because the quality is formed in this stage and control actions at the end of the production process cannot improve the quality of a product with poor quality of design [7].

The Taguchi robust design is a famous design procedure. It is an engineering method for optimizing the product or process condition to minimize the product sensitivity to the noise factors in the environment, such as: ambient temperature, humidity, air pressure and direct sunlight [8]. So, a product with high quality and low cost is being produced. One property of this approach is to investigate the quality characteristics numerically. In this approach the quality characteristics are grouped into three classes as: nominal the best (NTB), larger the better (LTB) and smaller the better (STB). Each of these quality characteristics could be constant or variable over time [9].

The target value and the specification limits for the time-oriented quality characteristics are being changed over time. So, for the design of a product with these quality characteristics, the parameters are designed such that the quality characteristics are being as close to their pre-specified target values as possible.

In this regard, three basic topics need to be introduced.

#### Design of experiments (DOE)

A collection of statistical methods that are used to find the influenced factors on a quality characteristic and to optimize its conditions. There are several types of DOE techniques including factorial experiments and fractional factorial experiments [10,11].

#### Response surface methodology (RSM)

A statistical and mathematical method for modeling, analyzing and optimizing the problems with response variables which are directly related to some other independent variables [12].

#### Desirability function

Is one of the common methods to simultaneously optimize multi response problems. The most applicable method of this type is the Derringer and Suich’s which is defined for several types of quality characteristics as follows [13]:

NTB quality characteristic:

(1) $$\mathrm{DF}\left(y\right)=\left\{\begin{array}{c} {\left[\frac{y-\mathrm{LSL}}{T-\mathrm{LSL}}\right]}^{r}\;,\;\mathrm{LSL}<y<T\\ {\left[\frac{y-\mathrm{USL}}{T-\mathrm{USL}}\right]}^{s}\;,\;T<y<\mathrm{USL}\\ \;0\;,\;y<\mathrm{LSL};y>\mathrm{USL} \end{array}\right)$$

LTB quality characteristic:

(2) $$\mathrm{DF}\left(y\right)=\left\{\begin{array}{c} \;1\;,\;y>y_{i}^{*}\\ {\left[\frac{y-y_{i_{*}}}{y_{i}^{*}-y_{i_{*}}}\right]}^{r}\;,\;y_{i_{*}}<y<y_{i}^{*}\\ \;0\;,\;y<y_{i_{*}} \end{array}\right)$$

STB quality characteristic

(3) $$\mathrm{DF}\left(y\right)=\left\{\begin{array}{c} \;1\;,\;y<y_{i_{*}}\\ {\left[\frac{y_{i}^{*}-y}{y_{i}^{*}-y_{i_{*}}}\right]}^{r}\;,\;y_{i_{*}}<y<y_{i}^{*}\\ \;0\;,\;y>y_{i}^{*} \end{array}\right)$$

In the above equations:

*y*: value observed for the quality characteristic

*T*: The target value for quality characteristic applicable for NTB quality characteristic.

*USL*: Upper specification Limit of NTB quality characteristic

*LSL*: Lower specification Limit of NTB quality characteristic

*y*_i_*: optimum point for LTB quality characteristic and highest acceptable value for STB quality characteristic

*y*_i*_: Optimum point for STB quality characteristic and lowest acceptable value for LTB quality characteristic

*r, s*: Weight values, positive constants.

### Problem definition

The drugs have a pre-determined profile for release based on the drug’s controlled-release mechanism. The aim in any drug laboratory is to find optimum adjustment of the controllable factors, such as material, production machine settings and so on to produce drugs that achieve the predetermined profile as much as possible. Four methods already exist for parameter design of a drug to achieve its pre-determined profile:

### Contour overlay method

This method is applied by Gohel and Amin [14] to find the optimal values to the Diclofenac Sodium formulation. The aim is to determine the suitable values for the three main controllable factors: stirring speed, concentration of CaCl_2_ and percentage of liquid paraffin, all of which influence the drug efficacy. The pre-determined profile of release is defined in advance. Then, the regression function of the drug release as a response variable and the above-mentioned control factors as independent variables is obtained by the least square method. For each point of time, the response is computed and compared to the pre-specified value. In this method, one variable is kept fixed and a two dimensional plot is used to find the optimal values.

The disadvantage of this method is that when the number of control factors increases, the efficiency of the method to introduce optimal values decreases.

### Profile selection

In situations where the profile properties are hard to identify, selection of the best profile is done by using the pre-defined indices. Two of these indices are f_1_ and f_2_ defined as:

(4) $$f_{1}=\frac{\sum_{t=1}^{n}\left|R_{t}-T_{t}\right|}{\sum_{t=1}^{n}R_{t}}*100$$

(5) $$f_{2}=50*\mathrm{Log}\left\{{\left[1+\frac{1}{n}\sum_{t=1}^{n}{\left(R_{t}-T_{t}\right)}^{2}\right]}^{-0.5}*100\right\}$$

Where:

R_t_: Percentage of drug release obtained from the reference formulation

T_t_: Percentage of drug release obtained from the test formulation

n: number of observations

The first index, f_1_ is defined as the dissimilarity index. As long as its value is small; the profile is close to the reference profile. The second index, f_2_ is defined as similarity index and when its value is large; the profile is near to the reference profile [15,16].

### MSE minimizing method

This method is applied in three articles. Truong et al. [17] used this method to determine the optimum values for control factors of a regenerative drug based on a profile of seven points.

Park et al. [18] used this method to investigate two quality characteristics separately for six and seven point profiles. Shin et al. [19] used this method to assess two quality characteristics separately for eight and eleven point profiles.

The first step in this method is to gather data and to calculate the basic statistics such as the mean and the variance. Then the RSM for these statistics are computed at each point of time. The optimal values for the control factors are obtained such that the following objective function is minimized.

(6) $$\mathrm{Minimize}\sum_{q=1}^{w}{\left(\hat{M}\left(x,t_{q}\right)-T_{q}\right)}^{2}+\sum_{q=1}^{w}\hat{v}\left(x,t_{q}\right)\;,\;S.t:x\in \Omega$$

Where:$\hat{M}\left(x,t_{q}\right)$: The mean of the responses at time t_q_.

$\hat{v}\left(x,t_{q}\right)$: The variance of the responses at time t_q_.

T_q_: The pre-specified target value for the response variable for the time q.

w: The number of points in time under study.

### Method of minimizing the total cost

This method is used by Goethals and Cho [20] and also the experiment of Gohel and Amin [14] on the Diclufenac Sodium is reassessed. The logic behind this method is to find the optimal values for control factors that minimize the following objective function:

(7) $$\begin{array}{ll} \mathrm{Minimize}\;E\left[\mathrm{TC}\right] & =\sum_{q=1}^{w}[\int_{\mathrm{LS}L_{q}}^{\mathrm{US}L_{q}}L\left[Y\left(q\right)\right].f[Y\left(q\right)].\mathrm{dY}\left(q\right)\\ & \;+\int_{-\infty }^{\mathrm{LS}L_{q}}NC_{q1}.f[y\left(q\right)]\mathrm{dY}\left(q\right)\\ & \;+\int_{\mathrm{US}L_{q}}^{+\infty }NC_{q2}.f[Y\left(q\right)]\mathrm{dY}\left(q\right)] \end{array}$$

Where:

LSL_q_ and USL_q_: are the lower and the upper specification limits for the quality characteristic, respectively.

f(y(q)): is the probability distribution function for response variable at time q.

NC_q1_ and NC_q2_: are the costs corresponding to being greater than USL and smaller than LSL, respectively.

L(y (q)): is the quality loss function for the quality characteristic within the acceptable region, but not on the target.

w: is the number of time points under study.

### The proposed method

The proposed method is a systematic and straightforward technique for determining the optimum values for the control factors for a drug. So that in a specified time interval, the drug release follows its premeditated profile. This method requires the following steps:

1. Determination of the drug release profile: Considering the kind of drug and its mechanism of release, the pharmaceutics design of the release profile of a drug by consulting the specialist physicians. To facilitate the comparison between the standard profile and the drug profile function, some points on time are considered and the experiments are run in these points. At each time point, the target value and the upper and the lower specification limits are determined. Selection of the number of points under study is based on the type of the drug and its life cycle in the patient’s body.

2. Determination of the experiment templates: In this stage, many controllable factors such as raw material and production factors for the drug under study are determined. Several combinations of these controllable factors are being tested by running the experiments. One important logic of the DOE is to find as much as information possible from the minimum number of experiments. For each combination of the factor levels at each time point some data is collected. Then, the data are organized based on the Table 1.The primary statistics such as the mean, the variance and the coefficient of variation for each time point and the covariance between observations in different time points are calculated. The computational formulas used to compute these statistics are as follows:

**Table 1:** **Experimental format **[20]

***Run***	***x***	***Y*** **(1)**	${\bar{y}}_{1}$	$s_{1}^{2}$	**…**	***Y*** **( *w *)**	${\bar{y}}_{w}$	$s_{w}^{2}$
1	Control factor settings	*y*_111_…*y*_11*m*_	${\bar{y}}_{11}$	$s_{11}^{2}$	…	*y*_*w*11_…*y*_*w*1*m*_	${\bar{y}}_{w1}$	$s_{w1}^{2}$
2		*y*_121_…*y*_12*m*_	${\bar{y}}_{12}$	$s_{12}^{2}$	…	*y*_*w*21_…*y*_*w*2*m*_	${\bar{y}}_{w2}$	$s_{w2}^{2}$
.		…	…	…	…	…	…	…
*r*		*y*_1*r*1_…*y*_1*rm*_	${\bar{y}}_{1r}$	$s_{1r}^{2}$	…	*y*_*wr*1_…*y*_*wrm*_	${\bar{y}}_{\mathrm{wr}}$	$s_{\mathrm{wr}}^{2}$
.		…	…	…	…	…	…	…
*n*		*y*_1*n*1_…*y*_1*nm*_	${\bar{y}}_{1n}$	$s_{1n}^{2}$	…	*y*_*wn*1_…*y*_*wnm*_	${\bar{y}}_{\mathrm{wn}}$	$s_{\mathrm{wn}}^{2}$

(8) $${\bar{y}}_{\mathrm{qr}}=\frac{\sum_{w=1}^{m}y_{\mathrm{qrw}}}{m}$$

(9) $$s_{\mathrm{qr}}^{2}=\frac{\sum_{w=1}^{m}{\left(y_{\mathrm{qrw}}-{\bar{y}}_{\mathrm{qr}}\right)}^{2}}{m-1}$$

(10) $${\left(\frac{s}{m}\right)}_{\mathrm{qr}}=\frac{s_{\mathrm{qr}}}{{\bar{y}}_{\mathrm{qr}}}$$

(11) $$s_{i,j}=\frac{\sum_{r=1}^{m}\left(y_{\mathrm{ipr}}-{\bar{y}}_{\mathrm{ip}}\right)\left(y_{\mathrm{jpr}}-{\bar{y}}_{\mathrm{jp}}\right)}{m-1}$$

3. Determination of the relationships among the statistics and the control factors: By using RSM technique, the relationships are defined. For the sake of simplicity and prevention of using data with several scales, the control factors are coded by linear relationships.

(12) $$\begin{array}{l} {\hat{\mu }}_{q}\left(x\right)=x{\hat{\beta }}_{\mathrm{\mu q}}\;,\;{\hat{\beta }}_{\mathrm{\mu q}}={\left(x^{'}x\right)}^{-1}x^{'}{\bar{y}}_{q}\;,\;x=\left[\begin{array}{ccc} 1 & \cdots & x_{1,k-1}\\ \vdots & \ddots & \vdots \\ 1 & \cdots & x_{n,k-1} \end{array}\right]\;\\ {\bar{y}}_{q}=\left[{\bar{y}}_{q1},{\bar{y}}_{q2},\ldots ,{\bar{y}}_{\mathrm{qn}}\right]' \end{array}$$

(13) $$\begin{array}{l} {\hat{s}}_{q}^{2}\left(x\right)=x{\hat{\beta }}_{s^{2}q}\;,\;{\hat{\beta }}_{s^{2}q}={\left(x^{'}x\right)}^{-1}x^{'}s_{q}^{2}\;,\;x=\left[\begin{array}{ccc} 1 & \cdots & x_{1,k-1}\\ \vdots & \ddots & \vdots \\ 1 & \cdots & x_{n,k-1} \end{array}\right]\;\\ s_{q}^{2}=\left[s_{q1}^{2},s_{q2}^{2},\ldots ,s_{\mathrm{qn}}^{2}\right]' \end{array}$$

(14) $$\begin{array}{l} {\left(\left(\hat{\frac{s}{m}}\right)\right)}_{q}\left(x\right)=x{\hat{\beta }}_{\left(s/m\right)}q\;,\;{\hat{\beta }}_{\left(s/m\right)}q=(x^{'}x){}^{\left(-1\right)}x^{'}(s/m){}_{q},\\ \;x=\left[\begin{array}{ccc} 1 & \cdots & x_{1,k-1}\\ \vdots & \ddots & \vdots \\ 1 & \cdots & x_{n,k-1} \end{array}\right]\;\\ {\left(\frac{s}{m}\right)}_{q}=[(\frac{s}{m}){}_{q1},(\frac{s}{m}){}_{q2},\ldots ,(\frac{s}{m}){}_{\mathrm{qn}}]{}^{'} \end{array}$$

(15) $$\begin{array}{l} {\hat{s}}_{i,j}\left(x\right)=x{\hat{\beta }}_{s_{i,j}}\;,{\hat{\beta }}_{s_{i,j}}={\left(x^{'}x\right)}^{-1}x^{'}s_{i,j},\;x=\left[\begin{array}{ccc} 1 & \cdots & x_{1,k-1}\\ \vdots & \ddots & \vdots \\ 1 & \cdots & x_{n,k-1} \end{array}\right]\;\\ \;s_{i.j}=\left[s_{i,j,1},s_{i,j,2},\ldots ,s_{i,j,n}\right]' \end{array}$$

In the interest of time and cost, the number of control factors is reduced before running the experiments by using any technique such as screening experiments, as well as the forward, backward and stepwise regression.

4 Model optimization: Using the desirability function method, the optimal values for control factors are determined based on the type of quality characteristics and their specification limits such that their values come as close to the target values as possible. The desirability function of interest is:

(16) $$\begin{array}{ll} \mathrm{Maximize}D_{\mathrm{total}} & =\;\left\{\left[\prod_{i=1}^{n}D{\left(\mu_{i}\right)}^{w_{i}}\right].\left[\prod_{i=1}^{n}D{\left(s_{i}^{2}\right)}^{w_{i}^{'}}\right]\right)\\ & \;\left(.\left[\prod_{i=1}^{n}D{\left(\frac{s_{i}}{m_{i}}\right)}^{w_{i}^{''}}\right].\left[\prod_{i=1}^{n}D{\left(s_{i,j}\right)}^{w_{i}^{'''}}\right]\;\right\}\\ & \;\times \left(\frac{1}{\sum_{i=1}^{n}\left(w_{i}+w_{i}^{'}+w_{i}^{''}\right)+\sum_{i=1}^{\left(\begin{array}{l} n\\ 2 \end{array}\right)}w_{i}^{'''}}\right) \end{array}$$

The results are robust as long as the covariances between the observations for each pair of points are close to zero. So, when there is a deviation in some time intervals, they would not be transmitted to the other points.

The other advantage of the proposed method is its ability to be used for any part of the desirability function. For instance when we don’t have access to the entire data and only the mean and the variance of the observations are available, the covariance part of the model may be eliminated. Or if the mean of the observations at each point of time for different combinations is in hand, only the mean part of the model is being used. Also, by using the desirability function and its weighted values, one may use any indices in some points under study. For the sake of simplicity, in the examples provided in Section 5, equal weights are assigned to all statistical indices in all time periods.

### Numerical examples

To illustrate the applications of the proposed method, seven examples for different drugs are presented in this section adapted from credible pharmaceutical papers. These examples are solved by the proposed method to find the optimum values for the control factors of the drugs. The required material, the methods of pharmaceutical experiments and the data for each example are presented in the stated indicated references.

### Example 1

#### Diclufenac Sodium

The release profile of this drug is investigated by Gohel [14] and Goethals [20]. The contour overlay and the minimization of quality loss function methods are introduced in their papers, respectively. This drug has three main control factors given in Table 2.

**Table 2:** Main control factors influencing Diclufenac Sodium release

**Variable**	**Control factor**	**Level 1**	**Level 2**	**Level 3**
x_1_	Stirring speed (RPM)	500	1000	1500
x_2_	Concentration of calcium chloride	5%	10%	15%
x_3_	Percentage of liquid paraffin	0%	25%	50%

The first step is to code the control factors using the following relationships:

$$x_{1\left(\mathrm{new}\right)}=\frac{x_{1}-1000}{500},x_{2\left(\mathrm{new}\right)}=\frac{x_{2}-10}{5},x_{3\left(\mathrm{new}\right)}=\frac{x_{3}-25}{25}$$

In this research, three points of time for the drug release profile are being investigated with properties shown in Table 3.

**Table 3:** The target values and lower and upper values for example 1

**Response**	**Delay after usage**	**LSL**	**Target**	**USL**
y_1_	1 hour	20%	30%	40%
y_2_	6 hour	50%	60%	70%
y_3_	8 hour	65%	72.5%	80%

The response surface relationships for the mean, the variance, the coefficient of variation and the covariance between each pair of points under study are presented in the Appendix 1. Optimum values are shown in Table 4.

**Table 4:** Optimum values for example 1

**Variable**	**Control factor**	**Coded value**	**Uncoded value**
**x**_**1**_	Stirring speed (RPM)	−0.7576	621.2 rpm
**x**_**2**_	Concentration of calcium chloride	−0.3939	8.0305%
**x**_**3**_	Percentage of liquid paraffin	1	50%

### Example 2

#### Terazosin HCl dehydrate

The release profile for this drug is investigated by Shin [19] and the problem is solved by the MSE minimization method. This experiment has ten control factors as shown in Table 5.

**Table 5:** Control factors influencing Terazosin HCl dehydrate release

**Variable**	**Control factor**	**Level 1**	**Level 2**	**Level 3**	**Level 4**	**Level 5**
x_1_	PEO	93.71	100.77	107.77	171.04	234.31
x_2_	LH-11	0	7.03	14.06	77.33	140.6
x_3_	Syloid	0	7.03	14.06	77.33	140.6
x_4_	Ac-Di-Sol	0	7.03	14.06	77.33	140.6
x_5_	Na-CMC	0	7.03	14.06	77.33	140.6
x_6_	HEC	0	7.03	14.06	77.33	140.6
x_7_	NaH_2_PO_4_	0	7.03	14.06	77.33	140.6
x_8_	Citric acid	0	7.03	14.06	77.33	140.6
x_9_	Pharma coat 603	0	7.03	14.06	77.33	140.6
x_10_	Polyox N10	0	7.03	14.06	77.33	140.6

Noticing the large number of control factors in this example, five control factors x_1_, x_3_, x_7_, x_8_ and x_10_ are identified as significant control factors by using the stepwise regression method. The control factors are coded by the following relationships:

$$x_{i-\mathrm{new}}=\left\{\begin{array}{c} \frac{x_{i}-93.71}{7.03},i=1\\ \frac{x_{i}}{7.03},i=2,3,\ldots ,10 \end{array}\right)$$

In this research, 11 points of time of drug release profile are being investigated as presented in Table 6.

**Table 6:** The target values and lower and upper values for example 2

	**y**_**1**_	**y**_**2**_	**y**_**3**_	**y**_**4**_	**y**_**5**_	**y**_**6**_	**y**_**7**_	**y**_**8**_	**y**_**9**_	**y**_**10**_	**y**_**11**_
Time	0.5 h	1 h	1.5 h	2 h	3 h	4 h	6 h	8 h	10 h	12 h	24 h
LSL	4.8	8.8	10.24	12.88	18.08	23.84	34.8	41.12	48.24	54.8	65.84
Target	6	11	12.8	16.1	22.6	29.8	43.5	51.4	60.3	68.5	82.3
USL	7.2	13.2	15.36	19.32	27.12	35.76	52.2	61.68	72.36	82.2	98.76

The response surface relationships for the mean and the variance of the underlying data are presented in Appendix 2. Optimum values for this example are shown in Table 7.

**Table 7:** Optimum values for control factors for example 2

**Variable**	**Control factor**	**Coded value**	**Uncoded value**
x_1_	PEO	15.556	203.069
x_3_	Syloid	0.691	4.858
x_7_	NaH_2_PO_4_	14.748	103.675
X_8_	Citric acid	0	0
X_10_	Polyox N10	20	140.6

### Example 3

#### Verapamil HCl

The release profile of this drug is investigated by Siva [21]. The three main control factors for this drug are presented in Table 8.

**Table 8:** Main control factors influencing Verapamil HCl release

**Variable**	**Control factor**	**Level 1**	**Level 2**	**Level 3**
x_1_	Coating weigh gain	8%	11%	14%
x_2_	Duration of coating	24 h	36 h	48 h
x_3_	Amount of plasticizer	60%	90%	120%

The control factors are coded by the following relationships:

$$x_{1\left(\mathrm{new}\right)}=\frac{x_{1}-11}{3},x_{2\left(\mathrm{new}\right)}=\frac{x_{2}-36}{12},x_{3\left(\mathrm{new}\right)}=\frac{x_{3}-90}{30}$$

In this research, five points of time are investigated from release profile as shown in Table 9.

**Table 9:** The target values and lower and upper values for example 3

	**y**_**1**_	**y**_**2**_	**y**_**3**_	**y**_**4**_	**y**_**5**_
Time	2 h	4 h	6 h	9 h	12 h
LSL	13.36%	26.64%	40%	50%	80%
Target	16.7%	33.3%	50%	75%	100%
USL	20.04%	39.96%	60%	90%	120%

The RSM relationships for the mean, the variance and the coefficient of variation for the points in Table 8 are presented in Appendix 3. By using the desirability function method the optimum values obtained for control factors are shown in Table 10.

**Table 10:** Optimum values for control factors for example 3

**Variable**	**Control factor**	**Coded value**	**Uncoded value**
x_1_	Coating weigh gain	−0.6566	9.0302
x_2_	Duration of coating	0.5152	29.8176
x_3_	Amount of plasticizer	1	120

### Example 4

#### Metformin

The release profile for this drug is investigated by Nagrava [22]. The three main control factors are defined for this drug release as shown in Table 11.

**Table 11:** Main control factors for example 3

**Variable**	**Control factor**	**Level 1**	**Level 2**	**Level 3**
x_1_	Concentration of sodium alginate	1.25%	1.75%	2.25%
x_2_	Concentration of gellan gum	0%	0.25%	0.5%
x_3_	Concentration of metformin	2.5%	3.75%	5%

The values of the control factors are coded using the following relationships:

$$x_{1\left(\mathrm{new}\right)}=\frac{x_{1}-1.758}{1.25},x_{2\left(\mathrm{new}\right)}=\frac{x_{2}-0.25}{0.25},x_{3\left(\mathrm{new}\right)}=\frac{x_{3}-3.75}{1.25}$$

The three points of time for the release profile are investigated in this research have the properties provided in Table 12.

**Table 12:** The target values and lower and upper values for example 4

**Response**	**Delay after usage**	**LSL**	**Target**	**USL**
y_1_	0.5 hour	21%	23.5%	26%
y_2_	3.5 hours	62%	63.5%	65%
y_3_	8 hours	91%	92.5%	94%

The RSM relationships for the mean, the variance and the coefficient of variation for the data are presented in Appendix 4. By using the desirability function method the optimum values obtained for control factors are shown in Table 13.

**Table 13:** Optimum values of control factors for example 4

**Variable**	**Control factor**	**Coded value**	**Uncoded value**
x_1_	Concentration of sodium alginate	1	2.25%
x_2_	Concentration of gellan gum	−0.9192	0.0202%
x_3_	Concentration of metformin	−1	2.5%

### Example 5

#### Rhinetedin

The release profile of this drug is investigated by Patel [23]. The two main control factors for this drug are presented in Table 14.

**Table 14:** Main control factors for example 5

**Variable**	**Control factor**	**Level 1**	**Level 2**	**Level 3**
x_1_	Amount of gelucire 43/01	504	672	840
x_2_	Amount of ethylcellulose	84	168	252

The control factors are coded by the following relationships:

$$x_{1\left(\mathrm{new}\right)}=\frac{x_{1}-672}{168},x_{2\left(\mathrm{new}\right)}=\frac{x_{2}-168}{84}\;$$

In this research three time points are investigated from release profile are shown in Table 15.

**Table 15:** The target values and lower and upper specifications for example 5

**Response**	**Delay after usage**	**LSL**	**Target**	**USL**
y_1_	1 hour	26%	32.5%	39%
y_2_	5 hours	54%	67.5%	81%
y_3_	10 hours	68%	85%	102%

In this example, the index f_2_ is the measure of similarity between the drug release profile and the target profile. The RSM relationships are presented in Appendix 5 and the optimum values are shown in Table 16.

**Table 16:** Optimum values for example 5

**Variable**	**Control factor**	**Coded value**	**Uncoded value**
x_1_	Amount of gelucire 43/01	−0.909	657.7288
x_2_	Amount of ethylcellulose	1	252

### Example 6

#### Metoprolol

The release profile for this drug is investigated by Gohel [24]. The two main control factors defined for this drug are shown in Table 17.

**Table 17:** Main control factors for example 7

**Variable**	**Control factor**	**Level 1**	**Level 2**	**Level 3**
x_1_	% of xanthan gum	20%	30%	40%
x_2_	% of Methocel	10%	20%	30%

The control factor values are coded by using the following relationships:

$$x_{1\left(\mathrm{new}\right)}=\frac{x_{1}-30}{10},x_{2\left(\mathrm{new}\right)}=\frac{x_{2}-20}{10}\;$$

The three points of time for the drug release profile are presented in Table 18.

**Table 18:** The target values and lower and upper specification limits for example 7

**Response**	**Delay after usage**	**LSL**	**Target**	**USL**
y_1_	1 hour	15%	17.5%	20%
y_2_	4 hours	20%	30%	40%
y_3_	12 hours	60%	65%	70%
t_50_	-	6 h	7 h	8 h
MDT	-	8 h	9 h	10 h

In the study of this drug, f_2_, t_50_ (the time required for 50% of drug to be released) and mean dissolution time (MDT) are the measures of the similarity factor between release profile and the predefined profile, the time required to dissolve half of the drug and the mean dissolution time, respectively. The RSM relationships for the means and these measures are presented in Appendix 6. By using the proposed method, the optimum values are obtained as shown in Table 19.

**Table 19:** Optimum values for example 7

**Variable**	**Control factor**	**Coded value**	**Uncoded value**
x_1_	% of xanthan gum	0.0458	30.458
x_2_	% of Methocel	0.6726	26.726

### Comparison of the proposed method and the existing ones

The disadvantages of the existing methods are:

Contour overlay method:

This method has a limited application and when the number of variables exceeds from two, the model may not be optimized unless the additional variables are being fixed at a constant level.

Profile selection method:

In this method, the number of test profiles is adjusted based on the experimenter point of view and the best profile is selected among the existing ones. It is possible that the optimum values for the control factors may not be included in these profiles.

MSE minimizing method:

In this method, there is no attention paid to the specification limits, while in the real world, passing these limits has substantial penalties.

Minimizing the total cost method:

In this method all deviations from the target values are evaluated by means of money terms, while in human problems, e.g. pharmaceutical studies, adverse events may have human fallout which cannot be measured by money terms.

The proposed method overcomes all the above disadvantages.

## Conclusions

Investigation of the pharmaceutics problems in an industrial engineering framework is very constructive. The key point here is the problem presentation by the engineering terms. In this research, the drug release problem which is an important subject of pharmaceutics is being studied. In this area, applying the complex formulas is avoided. So, the experts with minimum knowledge of mathematics and statistics may apply this approach to solve the pharmaceutics problems. The results of the examples show the ability of the proposed model for solving the controlled release problems and to assure that the intended drug is resolved as its predefined profile. The simultaneous optimization of drugs with multi time-oriented quality characteristics is a topic for the future research.

## Appendix 1

$$\begin{array}{ll} \mu_{1\left(1h\right)} & =39.929+2.365x_{1}-2.206x_{2}-1.959x_{3}\\ & \;+0.202x_{1}^{2}+1.971x_{2}^{2}-0.912x_{3}^{2}-1.389x_{1}x_{2}\\ & \;+0.797x_{1}x_{3}+0.079x_{2}x_{3} \end{array}$$

$$\begin{array}{ll} \mu_{2\left(6h\right)} & =73.368+4.388x_{1}-5.031x_{2}-2.379x_{3}\\ & \;+0.399x_{1}^{2}+0.579x_{2}^{2}-0.127x_{3}^{2}-1.525x_{1}x_{2}\\ & \;-0.062x_{1}x_{3}-0.359x_{2}x_{3} \end{array}$$

$$\begin{array}{ll} \mu_{3\left(8h\right)} & =83.203+4.165x_{1}-4.562x_{2}-2.498x_{3}\\ & \;-0.624x_{1}^{2}-0.907x_{2}^{2}+1.176x_{3}^{2}-2.37x_{1}x_{2}\\ & \;+0.151x_{1}x_{3}-1.632x_{2}x_{3} \end{array}$$

$$\begin{array}{ll} V_{1\left(1h\right)} & =7.31-0.642x_{1}+0.032x_{2}+2.799x_{3}\\ & \;+1.698x_{1}^{2}+5.377x_{2}^{2}+4.895x_{3}^{2}+5.543x_{1}x_{2}\\ & \;+1.893x_{1}x_{3}-0.686x_{2}x_{3} \end{array}$$

$$\begin{array}{ll} V_{2\left(6h\right)} & =5.74-1.195x_{1}+1.609x_{2}-5.458x_{3}\\ & \;+7.112x_{1}^{2}+0.037x_{2}^{2}+9.608x_{3}^{2}+11.9x_{1}x_{2}\\ & \;-4.042x_{1}x_{3}+0.98x_{2}x_{3} \end{array}$$

$$\begin{array}{ll} V_{3\left(8h\right)} & =11.548-6.216x_{1}+3.632x_{2}-0.354x_{3}\\ & \;+2.053x_{1}^{2}+2.293x_{2}^{2}+2.581x_{3}^{2}-5.282x_{1}x_{2}\\ & \;+2.575x_{1}x_{3}-5.902x_{2}x_{3} \end{array}$$

$$\begin{array}{ll} {\left(\frac{s}{m}\right)}_{1\left(1h\right)} & =0.063-0.005x_{1}-0.001x_{2}+0.012x_{3}\\ & \;+0.007x_{1}^{2}+0.008x_{2}^{2}+0.014x_{3}^{2}\\ & \;+0.021x_{1}x_{2}+0.008x_{1}x_{3}-0.003x_{2}x_{3} \end{array}$$

$$\begin{array}{ll} {\left(\frac{s}{m}\right)}_{2\left(6h\right)} & =0.04-0.002x_{1}+0.007x_{2}-0.003x_{3}\\ & \;+0.006x_{1}^{2}-0.004x_{2}^{2}+0.009x_{3}^{2}\\ & \;+0.013x_{1}x_{2}-0.008x_{1}x_{3}+0.004x_{2}x_{3} \end{array}$$

$$\begin{array}{ll} {\left(\frac{s}{m}\right)}_{3\left(8h\right)} & =0.039-0.009x_{1}+0.008x_{2}+0.002x_{3}\\ & \;-0.0002x_{1}^{2}+0.004x_{2}^{2}+0.006x_{3}^{2}-0.007x_{1}x_{2}\\ & \;+0.002x_{1}x_{3}-0.006x_{2}x_{3} \end{array}$$

$$\begin{array}{ll} {\left(s_{12}\right)}_{\left(1h-6h\right)} & =1.89+2.507x_{1}-0.799x_{2}+0.299x_{3}\\ & \;+0.677x_{1}^{2}-2.227x_{2}^{2}-4.571x_{3}^{2}-2.594x_{1}x_{2}\\ & \;-0.655x_{1}x_{3}-2.289x_{2}x_{3} \end{array}$$

$$\begin{array}{ll} {\left(s_{13}\right)}_{\left(1h-8h\right)} & =1.091+1.603x_{1}1.572x_{2}+3.023x_{3}\\ & \;-3.872x_{1}^{2}-3.299x_{2}^{2}+3.353x_{3}^{2}-1.879x_{1}x_{2}\\ & \;+0.966x_{1}x_{3}+2.559x_{2}x_{3} \end{array}$$

$$\begin{array}{ll} {\left(s_{23}\right)}_{\left(6h-8h\right)} & =-2.945-1.711x_{1}-1.729x_{2}-3.296x_{3}\\ & \;+2.541x_{1}^{2}+2.411x_{2}^{2}-2.738x_{3}^{2}-0.22x_{1}x_{2}\\ & \;+3.237x_{1}x_{3}+3.732x_{2}x_{3} \end{array}$$

## Appendix 2

$$\begin{array}{ll} \mu_{1\left(0.5h\right)} & =4.844-0.039x_{1}+0.023x_{3}-0.006x_{7}-0.005x_{8}\\ & \;-0.001x_{10}+0.0001x_{1}^{2}-0.00007x_{3}^{2}+0.00006x_{7}^{2}\\ & \;+0.00002x_{8}^{2}+0.00003x_{10}^{2}+0.0006x_{1}x_{3} \end{array}$$

$$\begin{array}{ll} V_{1\left(0.5h\right)} & =0.71-0.008x_{1}+0.0001x_{3}-0.00078x_{7}\\ & \;+0.006x_{8}-0.006x_{10}+0.00003x_{1}^{2}\\ & \;+0.000006x_{3}^{2}+0.00003x_{7}^{2}-0.00002x_{8}^{2}\\ & \;+0.00004x_{10}^{2}-0.00003x_{1}x_{3} \end{array}$$

$$\begin{array}{ll} \mu_{2\left(1h\right)} & =7.644-0.027x_{1}+0.015x_{3}-0.01x_{7}+0.017x_{8}\\ & \;-0.014x_{10}+0.0001x_{1}^{2}+0.000001x_{3}^{2}+0.0001x_{7}^{2}\\ & \;+0.0002x_{8}^{2}+0.0001x_{10}^{2}+0.0004x_{1}x_{3} \end{array}$$

$$\begin{array}{ll} V_{2\left(1h\right)} & =1.103-0.041x_{1}-0.027x_{3}-0.002x_{7}\\ & \;+0.021x_{8}+0.006x_{10}+0.0001x_{1}^{2}\\ & \;+0.00008x_{3}^{2}+0.00002x_{7}^{2}-0.00007x_{8}^{2}\\ & \;-0.00002x_{10}^{2}+0.0009x_{1}x_{3} \end{array}$$

$$\begin{array}{ll} \mu_{3\left(1.5h\right)} & =7.228+0.109x_{1}+0.018x_{3}-0.029x_{7}+0.033x_{8}\\ & \;-0.035x_{10}-0.0003x_{1}^{2}-0.0005x_{3}^{2}+0.0003x_{7}^{2}\\ & \;+0.0003x_{8}^{2}+0.0002x_{10}^{2}-0.0044x_{1}x_{3} \end{array}$$

$$\begin{array}{ll} V_{3\left(1.5h\right)} & =0.292+0.021x_{1}+0.035x_{3}-0.031x_{7}\\ & \;+0.033x_{8}-0.004x_{10}-0.00005x_{1}^{2}-0.000009x_{3}^{2}\\ & \;+0.0002x_{7}^{2}-0.0001x_{8}^{2}+0.00003x_{10}^{2}-0.0009x_{1}x_{3} \end{array}$$

$$\begin{array}{ll} \mu_{4\left(2h\right)} & =8.611+0.165x_{1}+0.248x_{3}-0.074x_{7}\\ & \;+0.074x_{8}-0.05x_{10}-0.0005x_{1}^{2}-0.0007x_{3}^{2}\\ & \;+0.0006x_{7}^{2}+0.0002x_{8}^{2}+0.0003x_{10}^{2}-0.006x_{1}x_{3} \end{array}$$

$$\begin{array}{ll} V_{4\left(2h\right)} & =1.582-0.082x_{1}-0.05x_{3}-0.033x_{7}+0.058x_{8}\\ & \;+0.027x_{10}+0.0003x_{1}^{2}+0.0002x_{3}^{2}\\ & \;+0.0002x_{7}^{2}-0.0002x_{8}^{2}-0.0001x_{10}^{2}+0.002x_{1}x_{3} \end{array}$$

$$\begin{array}{ll} \mu_{5\left(3h\right)} & =12.428+0.207x_{1}+0.309x_{3}-0.09x_{7}\\ & \;+0.089x_{8}-0.049x_{10}-0.0006x_{1}^{2}-0.0008x_{3}^{2}\\ & \;+0.0007x_{7}^{2}+0.0003x_{8}^{2}+0.0004x_{10}^{2}-0.008x_{1}x_{3} \end{array}$$

$$\begin{array}{ll} V_{5\left(3h\right)} & =1.69-0.078x_{1}-0.033x_{3}-0.021x_{7}+0.052x_{8}\\ & \;+0.033x_{10}+0.0003x_{1}^{2}+0.0001x_{3}^{2}\\ & \;+0.0001x_{7}^{2}-0.0002x_{8}^{2}-0.0001x_{10}^{2}+0.001x_{1}x_{3} \end{array}$$

$$\begin{array}{ll} \mu_{6\left(4h\right)} & =16.417+0.287x_{1}+0.388x_{3}-0.11x_{7}\\ & \;+0.126x_{8}-0.07x_{10}-0.0008x_{1}^{2}-0.001x_{3}^{2}\\ & \;+0.0009x_{7}^{2}+0.0003x_{8}^{2}+0.0005x_{10}^{2}-0.011x_{1}x_{3} \end{array}$$

$$\begin{array}{ll} V_{6\left(4h\right)} & =3.123-0.134x_{1}-0.074x_{3}-0.035x_{7}+0.061x_{8}\\ & \;+0.053x_{10}+0.0005x_{1}^{2}+0.0002x_{3}^{2}\\ & \;+0.0002x_{7}^{2}-0.0002x_{8}^{2}-0.0002x_{10}^{2}+0.003x_{1}x_{3} \end{array}$$

$$\begin{array}{ll} \mu_{7\left(6h\right)} & =21.874+0.563x_{1}+0.691x_{3}-0.174x_{7}\\ & \;+0.109x_{8}-0.084x_{10}-0.002x_{1}^{2}-0.002x_{3}^{2}\\ & \;+0.001x_{7}^{2}+0.0006x_{8}^{2}+0.0007x_{10}^{2}-0.02x_{1}x_{3} \end{array}$$

$$\begin{array}{ll} V_{7\left(6h\right)} & =4.719-0.22x_{1}-0.104x_{3}-0.056x_{7}+0.073x_{8}\\ & \;+0.105x_{10}+0.0008x_{1}^{2}+0.0003x_{3}^{2}\\ & \;+0.0003x_{7}^{2}-0.0002x_{8}^{2}-0.0004x_{10}^{2}+0.005x_{1}x_{3} \end{array}$$

$$\begin{array}{ll} \mu_{8\left(8h\right)} & =28.588+0.811x_{1}+0.963x_{3}-0.221x_{7}\\ & \;+0.073x_{8}-0.11x_{10}-0.002x_{1}^{2}-0.003x_{3}^{2}\\ & \;+0.001x_{7}^{2}+0.0007x_{8}^{2}+0.001x_{10}^{2}-0.03x_{1}x_{3} \end{array}$$

$$\begin{array}{ll} V_{8\left(8h\right)} & =5.417-0.226x_{1}-0.064x_{3}-0.072x_{7}+0.061x_{8}\\ & \;+0.158x_{10}+0.0008x_{1}^{2}+0.0001x_{3}^{2}\\ & \;+0.0004x_{7}^{2}-0.0002x_{8}^{2}-0.0006x_{10}^{2}+0.004x_{1}x_{3} \end{array}$$

$$\begin{array}{ll} \mu_{9\left(10h\right)} & =37.1+0.886x_{1}+1.086x_{3}-0.249x_{7}\\ & \;+0.058x_{8}-0.094x_{10}-0.003x_{1}^{2}-0.003x_{3}^{2}\\ & \;+0.002x_{7}^{2}+0.001x_{8}^{2}+0.001x_{10}^{2}-0.032x_{1}x_{3} \end{array}$$

$$\begin{array}{ll} V_{9\left(10h\right)} & =7.351-0.28x_{1}-0.085x_{3}-0.088x_{7}+0.046x_{8}\\ & \;+0.201x_{10}+0.001x_{1}^{2}+0.0002x_{3}^{2}\\ & \;+0.0005x_{7}^{2}-0.0002x_{8}^{2}-0.0008x_{10}^{2}+0.005x_{1}x_{3} \end{array}$$

$$\begin{array}{ll} \mu_{10\left(12h\right)} & =44.362+1.017x_{1}+1.237x_{3}-0.229x_{7}\\ & \;+0.055x_{8}-0.144x_{10}-0.003x_{1}^{2}-0.004x_{3}^{2}\\ & \;+0.001x_{7}^{2}+0.0006x_{8}^{2}+0.001x_{10}^{2}-0.036x_{1}x_{3} \end{array}$$

$$\begin{array}{ll} V_{10\left(12h\right)} & =7.482-0.267x_{1}-0.049x_{3}-0.095x_{7}+0.055x_{8}\\ & \;+0.217x_{10}+0.001x_{1}^{2}+0.00001x_{3}^{2}\\ & \;+0.0005x_{7}^{2}-0.0002x_{8}^{2}-0.001x_{10}^{2}+0.004x_{1}x_{3} \end{array}$$

$$\begin{array}{ll} \mu_{11\left(24h\right)} & =82.688+0.577x_{1}+0.705x_{3}-0.056x_{7}\\ & \;+0.06x_{8}+0.044x_{10}-0.002x_{1}^{2}-0.002x_{3}^{2}\\ & \;+0.004x_{7}^{2}-0.00004x_{8}^{2}+0.0001x_{10}^{2}-0.02x_{1}x_{3} \end{array}$$

$$\begin{array}{ll} V_{11\left(24h\right)} & =7.503-0.104x_{1}-0.025x_{3}-0.097x_{7}-0.005x_{8}\\ & \;-0.004x_{10}+0.0005x_{1}^{2}+0.00004x_{3}^{2}\\ & \;+0.0006x_{7}^{2}-0.0001x_{8}^{2}-0.0001x_{10}^{2}+0.001x_{1}x_{3} \end{array}$$

## Appendix 3

$$\begin{array}{ll} \mu_{1\left(2h\right)} & =12.986-2.16x_{1}-x_{2}+0.68x_{3}+0.121x_{1}^{2}-0.279x_{2}^{2}\\ & \;+0.221x_{3}^{2}+0.038x_{1}x_{2}+0.038x_{1}x_{3}+0.163x_{2}x_{3} \end{array}$$

$$\begin{array}{ll} v_{1\left(2h\right)} & =1.274+0.057x_{1}+0.33x_{2}-0.235x_{3}-0.064x_{1}^{2}\\ & \;+0.056x_{2}^{2}-0.298x_{3}^{2}-0.002x_{1}x_{2}+0.426x_{1}x_{3}\\ & \;+0.292x_{2}x_{3} \end{array}$$

$$\begin{array}{ll} {\left(\frac{s}{m}\right)}_{1\left(2h\right)} & =0.082+0.013x_{1}+0.017x_{2}-0.015x_{3}\\ & \;-0.006x_{1}^{2}+0.011x_{2}^{2}-0.016x_{3}^{2}+0.002x_{1}x_{2}\\ & \;+0.014x_{1}x_{3}+0.015x_{2}x_{3} \end{array}$$

$$\begin{array}{ll} \mu_{2\left(4h\right)} & =25.121-5.2x_{1}-2x_{2}+1.43x_{3}+0.47x_{1}^{2}-0.331x_{2}^{2}\\ & \;+0.619x_{3}^{2}+0.163x_{1}x_{2}+0.063x_{1}x_{3}+0.338x_{2}x_{3} \end{array}$$

$$\begin{array}{ll} v_{2\left(4h\right)} & =1.747+0.112x_{1}+0.004x_{2}-0.564x_{3}-1.017x_{1}^{2}\\ & \;+1.813x_{2}^{2}-0.732x_{3}^{2}-0.442x_{1}x_{2}\\ & \;+0.185x_{1}x_{3}-0.185x_{2}x_{3} \end{array}$$

$$\begin{array}{ll} {\left(\frac{s}{m}\right)}_{2\left(4h\right)} & =0.046+0.01x_{1}+0.002x_{2}-0.011x_{3}\\ & \;-0.013x_{1}^{2}+0.025x_{2}^{2}-0.009x_{3}^{2}-0.004x_{1}x_{2}\\ & \;+0.0004x_{1}x_{3}-0.004x_{2}x_{3} \end{array}$$

$$\begin{array}{ll} \mu_{3\left(6h\right)} & =42.938-7.27x_{1}-2.87x_{2}+2.31x_{3}\\ & \;-0.257x_{1}^{2}-0.257x_{2}^{2}-0.057x_{3}^{2}+0.913x_{1}x_{2}+0.463x_{1}x_{3}\\ & \;-0.688x_{2}x_{3} \end{array}$$

$$\begin{array}{ll} v_{3\left(6h\right)} & =3.412+0.072x_{1}+0.965x_{2}+0.052x_{3}\\ & \;+3.869x_{1}^{2}-1.106x_{2}^{2}-1.351x_{3}^{2}-1.126x_{1}x_{2}\\ & \;+0.936x_{1}x_{3}-0.049x_{2}x_{3} \end{array}$$

$$\begin{array}{ll} {\left(\frac{s}{m}\right)}_{3\left(6h\right)} & =0.042+0.009x_{1}+0.008x_{2}-0.002x_{3}\\ & \;-0.023x_{1}^{2}-0.005x_{2}^{2}-0.007x_{3}^{2}-0.006x_{1}x_{2}\\ & \;+0.004x_{1}x_{3}+0.001x_{2}x_{3} \end{array}$$

$$\begin{array}{ll} \mu_{4\left(9h\right)} & =67.278-11.37x_{1}-3.02x_{2}+3.27x_{3}-2.541x_{1}^{2}\\ & \;+3.709x_{2}^{2}-3.841x_{3}^{2}+0.125x_{1}x_{2}+0.825x_{1}x_{3}\\ & \;+0.05x_{2}x_{3} \end{array}$$

$$\begin{array}{ll} v_{4\left(9h\right)} & =3.563+0.311x_{1}-0.064x_{2}+0.085x_{3}-0.895x_{1}^{2}\\ & \;+0.32x_{2}^{2}+0.425x_{3}^{2}+0.523x_{1}x_{2}-0.208x_{1}x_{3}\\ & \;-0.09x_{2}x_{3} \end{array}$$

$$\begin{array}{ll} {\left(\frac{s}{m}\right)}_{4\left(9h\right)} & =0.027+0.007x_{1}+0.002x_{2}-0.002x_{3}\\ & \;-0.001x_{1}^{2}+0.001x_{2}^{2}+0.003x_{3}^{2}+0.003x_{1}x_{2}\\ & \;-0.002x_{1}x_{3}-0.001x_{2}x_{3} \end{array}$$

$$\begin{array}{ll} \mu_{5\left(12h\right)} & =82.395-12.84x_{1}-5.25x_{2}+3.8x_{3}-0.567x_{1}^{2}\\ & \;-0.417x_{2}^{2}+0.333x_{3}^{2}-0.675x_{1}x_{2}+0.625x_{1}x_{3}\\ & \;+0.125x_{2}x_{3} \end{array}$$

$$\begin{array}{ll} v_{5\left(12h\right)} & =3.944-0.428x_{1}+0.038x_{2}-0.142x_{3}+1.018x_{1}^{2}\\ & \;-1.592x_{2}^{2}-0.662x_{3}^{2}+0.705x_{1}x_{2}-0.065x_{1}x_{3}\\ & \;+0.643x_{2}x_{3} \end{array}$$

$$\begin{array}{ll} {\left(\frac{s}{m}\right)}_{5\left(12h\right)} & =0.024+0.002x_{1}+0.002x_{2}-0.002x_{3}\\ & \;+0.004x_{1}^{2}-0.006x_{2}^{2}-0.002x_{3}^{2}+0.003x_{1}x_{2}\\ & \;-0.001x_{1}x_{3}+0.003x_{2}x_{3} \end{array}$$

## Appendix 4

$$\begin{array}{ll} \mu_{1\left(0.5h\right)} & =31.153-3.546x_{1}-3.884x_{2}+3.243x_{3}\\ & \;+0.667x_{1}^{2}+1.874x_{2}^{2}-3.391x_{3}^{2}\\ & \;+2.897x_{1}x_{2}-0.767x_{1}x_{3}+1.175x_{2}x_{3} \end{array}$$

$$\begin{array}{ll} v_{1\left(0.5h\right)} & =0.669-0.456x_{1}-0.45x_{2}-0.839x_{3}+1.542x_{1}^{2}\\ & \;-1.429x_{2}^{2}+2.026x_{3}^{2}-1.309x_{1}x_{2}-1.167x_{1}x_{3}\\ & \;+0.649x_{2}x_{3} \end{array}$$

$$\begin{array}{ll} {\left(\frac{s}{m}\right)}_{1\left(0.5h\right)} & =0.028+0.002x_{1}+0.0004x_{2}-0.016x_{3}\\ & \;+0.01x_{1}^{2}-0.01x_{2}^{2}+0.022x_{3}^{2}-0.016x_{1}x_{2}\\ & \;-0.01x_{1}x_{3}+0.002x_{2}x_{3} \end{array}$$

$$\begin{array}{ll} \mu_{2\left(3.5h\right)} & =64.474-6.603x_{1}-4.648x_{2}+3.1x_{3}-0.977x_{1}^{2}\\ & \;+4.658x_{2}^{2}+1.287x_{3}^{2}-1.168x_{1}x_{2}-0.65x_{1}x_{3}\\ & \;-0.705x_{2}x_{3} \end{array}$$

$$\begin{array}{ll} v_{2\left(3.5h\right)} & =0.841-0.063x_{1}+0.215x_{2}+0.12x_{3}-0.173x_{1}^{2}\\ & \;+0.765x_{2}^{2}+0.048x_{3}^{2}-0.084x_{1}x_{2}-0.56x_{1}x_{3}\\ & \;-0.371x_{2}x_{3} \end{array}$$

$$\begin{array}{ll} {\left(\frac{s}{m}\right)}_{2\left(3.5h\right)} & =0.011+0.001x_{1}+0.003x_{2}-0.001x_{3}\\ & \;-0.001x_{1}^{2}+0.007x_{2}^{2}-0.0003x_{3}^{2}-0.00003x_{1}x_{2}\\ & \;-0.004x_{1}x_{3}-0.002x_{2}x_{3} \end{array}$$

$$\begin{array}{ll} \mu_{3\left(8h\right)} & =92.466-4.383x_{1}-2.878x_{2}+1.811x_{3}-1.242x_{1}^{2}\\ & \;+2.206x_{2}^{2}-0.987x_{3}^{2}-1.1x_{1}x_{2}+0.168x_{1}x_{3}\\ & \;+2.018x_{2}x_{3} \end{array}$$

$$\begin{array}{ll} v_{3\left(8h\right)} & =0.895-0.192x_{1}+0.213x_{2}-0.302x_{3}\\ & \;+0.029x_{1}^{2}-0.564x_{2}^{2}+0.786x_{3}^{2}-0.135x_{1}x_{2}\\ & \;+0.088x_{1}x_{3}-0.284x_{2}x_{3} \end{array}$$

$$\begin{array}{ll} {\left(\frac{s}{m}\right)}_{3\left(8h\right)} & =0.01-0.001x_{1}+0.001x_{2}-0.001x_{3}\\ & \;+0.0001x_{1}^{2}-0.004x_{2}^{2}+0.004x_{3}^{2}-0.001x_{1}x_{2}\\ & \;+0.001x_{1}x_{3}-0.001x_{2}x_{3} \end{array}$$

## Appendix 5

$$\begin{array}{ll} \mu_{1\left(1h\right)} & =37.191-7.918x_{1}-3.955x_{2}+1.148x_{1}^{2}-1.432x_{2}^{2}\\ & \;-0.558x_{1}x_{2} \end{array}$$

$$\begin{array}{ll} v_{1\left(1h\right)} & =1.957+0.862x_{1}-0.693x_{2}-0.105x_{1}^{2}-0.04x_{2}^{2}\\ & \;-1.32x_{1}x_{2} \end{array}$$

$$\begin{array}{ll} {\left(\frac{s}{m}\right)}_{1\left(1h\right)} & =0.038+0.015x_{1}-0.003x_{2}-0.002x_{1}^{2}-0.001x_{2}^{2}\\ & \;-0.012x_{1}x_{2} \end{array}$$

$$\begin{array}{ll} \mu_{2\left(5h\right)} & =75.29-6.358x_{1}-8.795x_{2}+1.035x_{1}^{2}-1.345x_{2}^{2}\\ & \;+0.745x_{1}x_{2} \end{array}$$

$$\begin{array}{ll} v_{2\left(5h\right)} & =5.129+0.25x_{1}+0.915x_{2}-2.223x_{1}^{2}-0.583x_{2}^{2}\\ & \;-1.18x_{1}x_{2} \end{array}$$

$$\begin{array}{ll} {\left(\frac{s}{m}\right)}_{2\left(5h\right)} & =0.031+0.003x_{1}+0.006x_{2}-0.009x_{1}^{2}\\ & \;-0.002x_{2}^{2}-0.005x_{1}x_{2} \end{array}$$

$$\begin{array}{ll} \mu_{3\left(10h\right)} & =89.216-8.49x_{1}-7.528x_{2}+3.797x_{1}^{2}-1.728x_{2}^{2}\\ & \;-3.195x_{1}x_{2} \end{array}$$

$$\begin{array}{ll} v_{3\left(10h\right)} & =3.026-0.145x_{1}-1.292x_{2}+2.372x_{1}^{2}-1.968x_{2}^{2}\\ & \;+0.75x_{1}x_{2} \end{array}$$

$$\begin{array}{ll} {\left(\frac{s}{m}\right)}_{3\left(10h\right)} & =0.017+0.002x_{1}-0.003x_{2}+0.007x_{1}^{2}\\ & \;-0.004x_{2}^{2}-0.003x_{1}x_{2} \end{array}$$

$$\begin{array}{ll} f_{2}\mathrm{value} & =50.157+7.52x_{1}+9.473x_{2}-5.26x_{1}^{2}-1.49x_{2}^{2}\\ & \;-0.66x_{1}x_{2} \end{array}$$

## Appendix 6

$$\begin{array}{ll} \mu_{1\left(1h\right)} & =20.778-3.317x_{1}-4.017x_{2}+0.183x_{1}^{2}-0.917x_{2}^{2}\\ & \;-0.325x_{1}x_{2} \end{array}$$

$$\begin{array}{ll} \mu_{2\left(4h\right)} & =38.678-4.5x_{1}-5.7x_{2}+1.583x_{1}^{2}-1.467x_{2}^{2}\\ & \;-1.425x_{1}x_{2} \end{array}$$

$$\begin{array}{ll} \mu_{3\left(12h\right)} & =68.822-5.483x_{1}-5.5x_{2}+2.317x_{1}^{2}-1.333x_{2}^{2}\\ & \;+0.15x_{1}x_{2} \end{array}$$

$$\mu_{4\left(t_{50}\right)}=6.222+x_{1}+1.167x_{2}-0.333x_{1}^{2}+0.167x_{2}^{2}$$

$$\begin{array}{ll} \mu_{5\left(\mathrm{MDT}\right)} & =8.222+0.767x_{1}+0.933x_{2}-0.333x_{1}^{2}\\ & \;+0.267x_{2}^{2}-0.1x_{1}x_{2} \end{array}$$

$$\begin{array}{ll} \mu_{6\left(f2\right)} & =68.556+11.183x_{1}+11.45x_{2}-2.483x_{1}^{2}\\ & \;-3.583x_{2}^{2}-1.525x_{1}x_{2} \end{array}$$

## Abbreviations

MEC: Minimum effective concentration; MTC: Minimal toxic concentration; NTB: Nominal the best; LTB: Larger the better; STB: Smaller the better; DOE: Design of experiments; RSM: Response surface methodology; LSL: Lower specification limit; USL: Upper specification limit; MDT: Mean dissolution time.

## Competing interests

The authors declare that they have no competing interests.

## Authors’ contributions

Authors contributed to the manuscript according to their responsibility. MRN designed and carried out the study. HS was the dissertation supervisor. MS validated the findings and proofread the final version. All authors read and approved the final manuscript.
